# COVID-19 Patient Health Prediction Using Boosted Random Forest Algorithm

**DOI:** 10.3389/fpubh.2020.00357

**Published:** 2020-07-03

**Authors:** Celestine Iwendi, Ali Kashif Bashir, Atharva Peshkar, R. Sujatha, Jyotir Moy Chatterjee, Swetha Pasupuleti, Rishita Mishra, Sofia Pillai, Ohyun Jo

**Affiliations:** ^1^BCC of Central South University of Forestry and Technology, Changsha, China; ^2^Department of Computing and Mathematics, Manchester Metropolitan University, Manchester, United Kingdom; ^3^Department of Information Technology, G H Raisoni College of Engineering, Nagpur, India; ^4^School of Information Technology and Engineering, VIT University, Vellore, India; ^5^Department of Information Technology, Lord Buddha Education Foundation, Kathmandu, Nepal; ^6^School of Civil Engineering, Galgotias University, Greater Noida, India; ^7^Department of Electronics and Telecommunications Engineering, G H Raisoni College of Engineering, Nagpur, India; ^8^School of Civil Engineering, Galgotias University, Greater Noida, India; ^9^Department of Computer Science, College of Electrical and Computer Engineering, Chungbuk National University, Cheongju-si, South Korea

**Keywords:** COVID-19, healthcare analytics, patient data, infection, boosting, random forest classification

## Abstract

Integration of artificial intelligence (AI) techniques in wireless infrastructure, real-time collection, and processing of end-user devices is now in high demand. It is now superlative to use AI to detect and predict pandemics of a colossal nature. The Coronavirus disease 2019 (COVID-19) pandemic, which originated in Wuhan China, has had disastrous effects on the global community and has overburdened advanced healthcare systems throughout the world. Globally; over 4,063,525 confirmed cases and 282,244 deaths have been recorded as of 11th May 2020, according to the European Centre for Disease Prevention and Control agency. However, the current rapid and exponential rise in the number of patients has necessitated efficient and quick prediction of the possible outcome of an infected patient for appropriate treatment using AI techniques. This paper proposes a fine-tuned Random Forest model boosted by the AdaBoost algorithm. The model uses the COVID-19 patient's geographical, travel, health, and demographic data to predict the severity of the case and the possible outcome, recovery, or death. The model has an accuracy of 94% and a F1 Score of 0.86 on the dataset used. The data analysis reveals a positive correlation between patients' gender and deaths, and also indicates that the majority of patients are aged between 20 and 70 years.

## Introduction

The healthcare industry is a vast industry that requires real time collection and processing of medical data. Moreover, at the core of this industry lies the problem of data handling which requires real time prediction and dissemination of information to practitioners for quick medical attention. Major actors of this industry, such as physicians, vendors, hospitals, and health-based companies have attempted to collect, manage, and revive data with the aim of using it to enhance medical practices and for technological innovation. However, dealing with healthcare data has, of late, become a complex task due to the massive volume of the data, security issues, wireless network application incompetence, and the velocity at which it is increasing. Thus, to increase the efficiency, accuracy, and workflow healthcare industries need data analytics tools to manage such complex data.

Coronavirus disease 2019 (COVID-19) is a virus of the Corona virus family and the source of a respiratory illness outbreak throughout the world that originated in Wuhan, China. Studies (1–3) show that Covid-19 has clinical characteristics akin to the SARS-CoV. The dominant symptoms include fever and cough, while gastrointestinal symptoms are uncommon. In COVID-19 infected patients the absence of fever is more frequent than in patients infected by similar viruses, i.e., MERS Corona Virus (2%) and SARS Corona Virus (1%) (4); therefore, there is a possibility of non-febrile patients being missed by a surveillance mechanism with a primary focus on detecting fever (5). The initial patients infected by COVID-19, reportedly indicated an association with a large seafood and animal market in Wuhan that demonstrated an animal-to-person spread. Per contra, a burgeoning number of patients have not displayed any association with the animal markets, revealing the fact of human-to-human transmission of COVID-19. This pandemic has been declared a global health emergency and is spreading at an alarming rate (6). The origin of the virus in Wuhan, China has caused 175,694 deaths globally and has 2,544,792 active patients globally (7). With the stress on medical facilities, it is essential for governments and healthcare facilities to identify and treat cases that are most likely to survive, by so doing, judiciously utilizing the limited stock of medical resources and medications.

Artificial Intelligence (AI) has emerged as the breakthrough technology of the twenty-first century and has found multiple applications in fields from weather prediction, astronomical exploration, to autonomous systems (8). We note a few related works where AI has been applied for detection, prevention, and prediction to combat the COVID-19 pandemic. In Wang and Wong (9) researchers have implemented a Convolutional Neural Network based model to detect COVID-19 patients using CXR images. They used a pre-trained ImageNet and trained the model on an open source dataset of Chest X-Ray images (CXR). While Pal et al. (10) implemented a LSTM model to predict the country-specific risk of COVID-19, that relies on trends and weather data of a particular country to predict the probable spread of COVID-19 in that country. In Liu et al. (11) the AI practitioners applied ML to process internet activity, news reports, health organization reports, and media activity to predict the spread of the outbreak on the providence level in China (12). In Bayes and Valdivieso (13) the authors made use of the Bayesian approach to predict the number of deaths in Peru for 70 days in the future, using the empirical data from China. The authors in Beck et al. (14) applied Artificial intelligence to identify the commercially available drugs that could be used to treat COVID-19 patients. They used Bidirectional Encoder Representations from the Transformers (BERT) framework at the core of their model. In Tang et al. (15) the researchers implemented the random forest algorithm for severity analysis of COVID-19 patients using the Computed Tomography (CT) Scans. In Khalifa et al. (16) the authors proposed a Generative Adversarial Network based fine-tuned model for detecting pneumonia from Chest X-Ray scans, which is one of the symptoms of COVID-19 infection. In Sujatha et al. (17), authors proposed a method which could be helpful in predicting the stretch of COVID-2019, by performing linear regression, and the Multilayer perceptron and Vector autoregression model which could provide an expectation on the COVID-19 Kaggle information, to anticipate the epidemiological pattern of the disease and rate of COVID-2019 cases in India.

Kutia et al. (18) tried to break down client perspectives to eHealth applications in China and the eHealth framework in the Ukraine, which afterwards provided bits of knowledge and proposals for the improvement of an eHealth application (eZdorovya) for mainly health information benefits. Sultan et al. (19) presented a hybrid method that generates and facilitates Alzheimer patients to recall their memories. This egocentric video summary uses important people, objects, and medicines as tools in the realization of their method. Furthermore, an emerging tactile Internet-based nanonetwork that promises a new range of e-health applications has been proposed by Feng et al. (20). The authors use an information based transmit network that goes to an operator via the terahertz band. Finally, the authors in Jain and Chatterjee (21) presented an assortment of strategies intended to speak to, improve, and enable multi-disciplinary and multi-institutional ML to explore in healthcare informatics (22). Khamparia et al. (23) introduced a unique way of an internet of health things (IoHT)-driven deep learning structure for identification and arrangement of cervical cancer in Pap smear pictures, utilizing ideas of transfer learning. Waheed et al. (24) suggested a technique to produce manufactured chest X-ray (CXR) pictures by building up an Auxiliary Classifier Generative Adversarial Network (ACGAN) utilized model called CovidGAN. Sakarkar et al. (25) suggested a profound learning-based mechanized discovery and characterization model for fundus DR pictures.

This paper aims to fill the void of the traditional healthcare system, using machine learning (ML) algorithms to simultaneously process healthcare and travel data along with other parameters of COVID-19 positive patients, in Wuhan, to predict the most likely outcome of a patient, based on their symptoms, travel history, and the delay in reporting the case by identifying patterns from previous patient data. Our contribution includes:

- Processing of healthcare and travel data using machine learning algorithms in place of the traditional healthcare system to identify COVID infected person.
- This work compared multiple algorithms that are available for processing patient data and identified the Boosted Random Forest as the best method for processing data. Further, it executed a grid search to fine-tune the hyper parameters of the Boosted Random Forest algorithm to improve performance.
- Our work obliterates the need to re-compare existing algorithms for processing COVID-19 patient data.
- This work will enable researchers to further work on developing a solution that combines the processing of patient demographics, travel, and subjective health data with image data (scans) for better prediction of COVID-19 patient health outcomes.

The rest of the article is organized as follows: section Materials and Methods discusses the materials and methodology used in detail, along with the dataset description, data pre-processing, and the data analysis of the classification algorithms used. Section Results discusses the result of the experiment followed by further discussion in section Discussion. Section Conclusion and Future Work discusses the results and provides a conclusion and the future direction of the current work.

## Materials and Methods

The dependencies for the project include the following packages and libraries: Datetime, Numpy, Pandas, SciPy, Scikit Learn, and Matplotlib. The project has been implemented on the Google Colab platform using the CPU runtime. The CPU specifications for Google Colab are; model: 79, CPU Family: 6, model name: Intel(R) Xeon(R) CPU @ 2.20 GHz and cache size: 56,320 KB. The storage used is Google Drive.

### Dataset

The dataset used in this study was accessed from Kaggle as “Novel Corona Virus 2019 Dataset” (26). The dataset has been compiled from various sources including the World Health Organization and John Hopkins University. However, this dataset has been pre-processed further by us to meet the needs of this study. Table 1 presents the features of the data.

**Table 1:** Dataset description.

**Column**	**Description**	**Values (for categorical variables)**	**Type**
id	Patient Id	NA	Numeric
location	The location where the patient belongs to	Multiple cities located throughout the world	String, Categorical
country	Patient's native country	Multiple countries	String, Categorical
gender	Patient's gender	Male, Female	String, Categorical
age	Patient's age	NA	Numeric
sym_on	The date patient started noticing the symptoms	NA	Date
hosp_vis	Date when the patient visited the hospital	NA	Date
vis_wuhan	Whether the patient visited Wuhan, China	Yes (1), No (0)	Numeric, Categorical
from_wuhan	Whether the patient belonged to Wuhan, China	Yes (1), No (0)	Numeric, Categorical
death	Whether the patient passed away due to COVID-19	Yes (1), No (0)	Numeric, Categorical
Recov	Whether the patient recovered	Yes (1), No (0)	Numeric, Categorical
symptom1. symptom2, symptom3, symptom4, symptom5, symptom6	Symptoms noticed by the patients	Multiple symptoms noticed by the patients	String, Categorical

### Data Analysis

**Figure 1:**
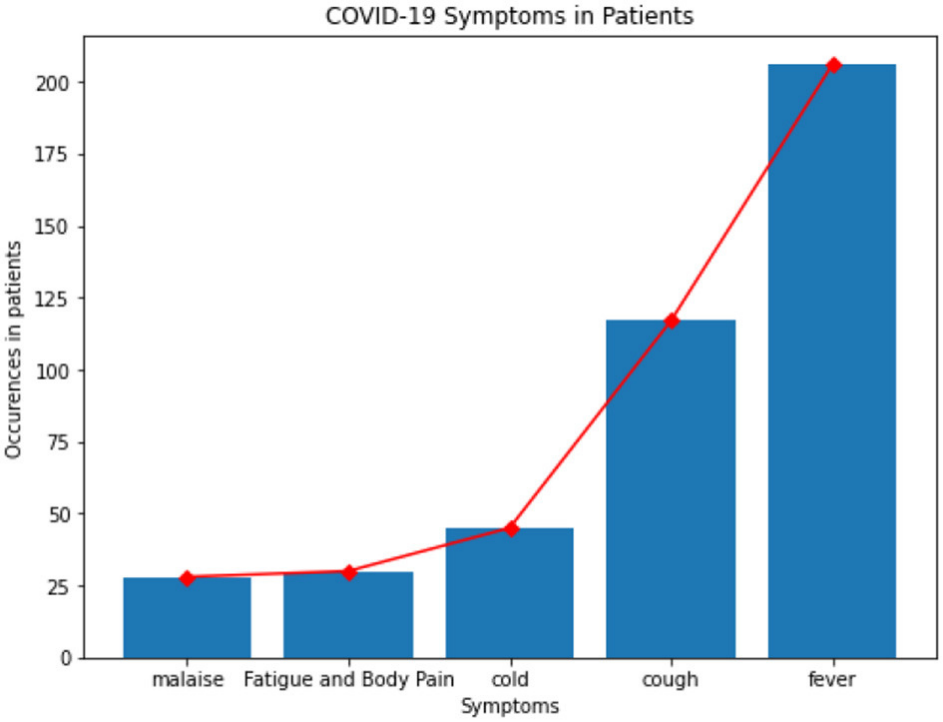
Symptoms in patients.

Fever, cough, cold, fatigue, body pain, and malaise were the most common symptoms that were noticed in patients whose data is available in this dataset and are shown in Figure 1.

**Figure 2:**
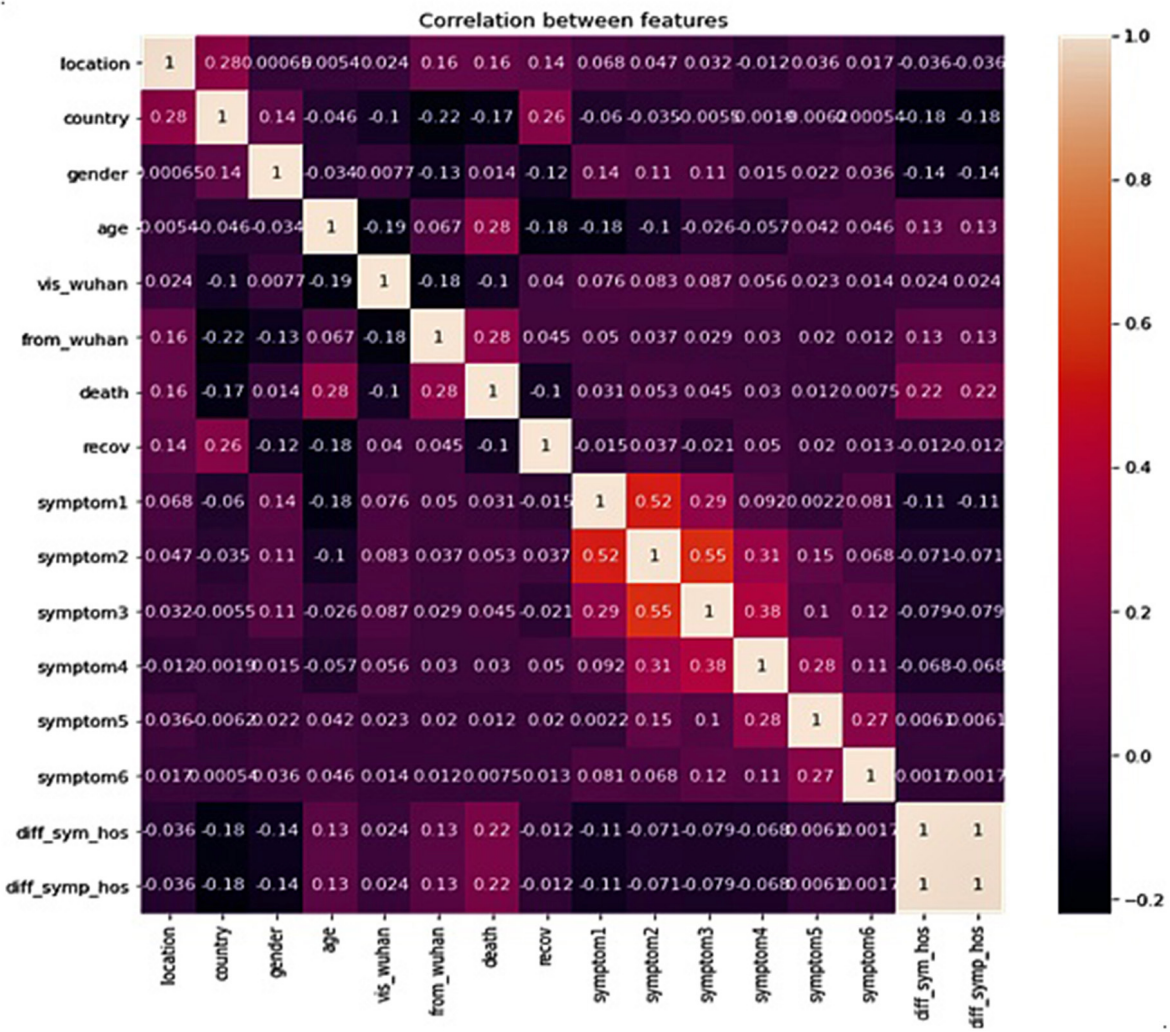
Correlation between data features.

Correlation between features of the dataset provides crucial information about the features and the degree of influence they have over the target value. The heat map of Pearson Correlation between the features of the dataset is shown in Figure 2, which clearly reveals a relatively stronger positive correlation between age of the patient, whether the patient was native to Wuhan, gap between (in days) when they first felt the symptoms and visited the hospital, and death. However, the country of the patient has a positive correlation with recovery. This implies that foreign patients who visited China had a higher recovery rate. There is also a strong positive correlation between symptom1 and symptom2, and also between symptom2 and symptom3.

### Data Pre-processing

The dataset consists of columns with the data being the Date, String, and Numeric type. We also have categorical variables in the dataset. Since the ML model requires all the data that is passed as input to be in the numeric form, we performed label-encoding of the categorical variables. This assigns a number to every unique categorical value in the column.

The dataset consists of multiple missing values which cause an error when passed directly as an input. Thus, we fill the missing values with “NA.” Certain patient data records contain missing values for both the “death” and “recov” columns, such patient records have been separated from the main dataset and compiled into the test dataset, while the remaining records have been compiled into the train dataset.

The dataset also consists of columns in the date format. Since the data columns are not directly used, feature engineering has been applied. A new column has been populated with the corresponding (hosp_vis—sym_on) value. This provides us with the number of days that have passed between the symptoms being noticed and the patient visiting the hospital.

### Evaluation Metrics

The purpose of the following study is to accurately predict the outcome of a particular patient depending on multiple factors, including but not limited to travel history, demographics etc. Since this is a very crucial prediction, accuracy is very important. Thus, for the purpose of evaluating the model we considered three evaluation metrics for this study.

The following terms are used in the equations: TP, True Positive; TN, True Negative; FP, False Positive; and FN, False Negative.

#### Accuracy

Given a dataset consisting of (*TP* + *TN*) data points, the accuracy is equal to the ratio of total correct predictions (*TP* + *TN* + *FP* + *FN*) by the classifier to the total data points. Accuracy is an important measure which is used to assess the performance of the classification model. Accuracy is calculated as shown in Equation (1) as follows:

(1) $$\begin{array}{r} \text{Accuracy}=\frac{\text{TP }+\text{ TN}}{\text{TP }+\text{ TN }+\text{ FP }+\text{ FN}}\;0.0<\text{Accuracy}<1.0 \end{array}$$

#### Precision

Precision is equal to the ratio of the True Positive (*TP*) samples to the sum of True Positive (*TP*) and False Positive (*FP*) samples. Precision is also a key metric to identify the number of correctly classified patients in an imbalanced class dataset. Precision is calculated as given in Equation (2) as follows:

(2) $$\begin{array}{r} \text{Precision}=\frac{\text{TP}}{\text{TP }+\text{ FP}} \end{array}$$

#### Recall

Recall is equal to the ratio of the True Positive (*TP*) samples to the sum of True Positive (*TP*) and False Negative (*FN*) samples. Recall is a significant metric to identify the number of correctly classified patients in an imbalanced class dataset out of all the patients that could have been correctly predicted. Recall is calculated as given in Equation (3) as follows:

(3) $$\begin{array}{r} \text{Recall}=\frac{\text{TP}}{\text{TP }+\text{ FN}} \end{array}$$

#### F1 Score

F1 Score is equal to the harmonic mean of Recall and Precision value. The F1 Score strikes the perfect balance between Precision and Recall thereby providing a correct evaluation of the model's performance in classifying COVID-19 patients. This is the most significant measure that we will be using to evaluate the model. F1 Score can be calculated as shown in Equation (4) as follows:

(4) $$\begin{array}{r} \text{F}1\text{ Score}=2\;\times \;\frac{\text{Precision }\times \text{ Recall}}{\text{Precision }+\text{ Recall}} \end{array}$$

## Results

We have used the pre-processed dataset to train multiple ML classification models. The models included in this study include: Decision Tree Classifier, Support Vector Classifier, Gaussian Naïve Bayes Classifier, and Boosted Random Forest Classifier.

**Figure 3:**
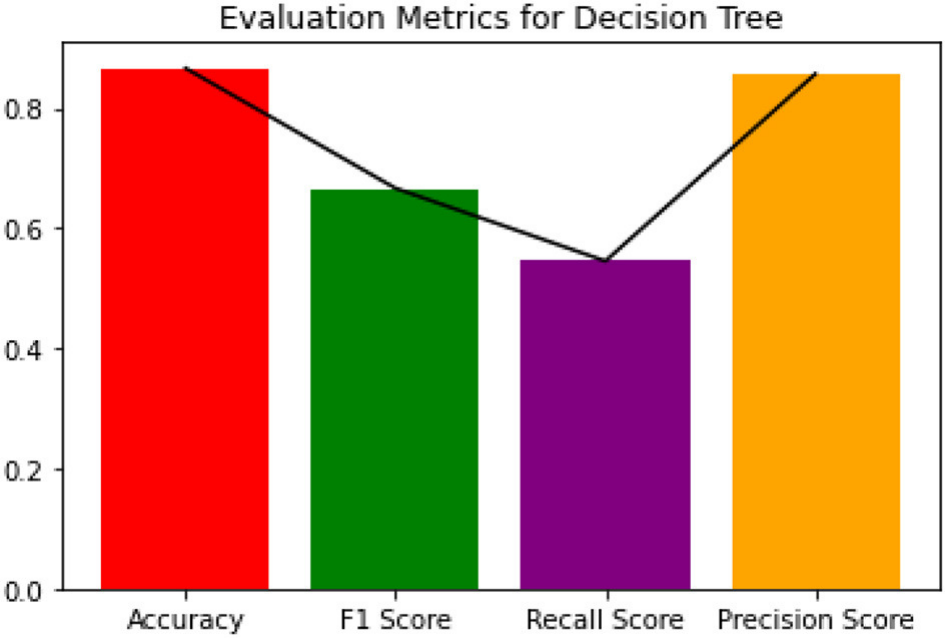
Evaluation metrics for decision tree.

**Figure 4:**
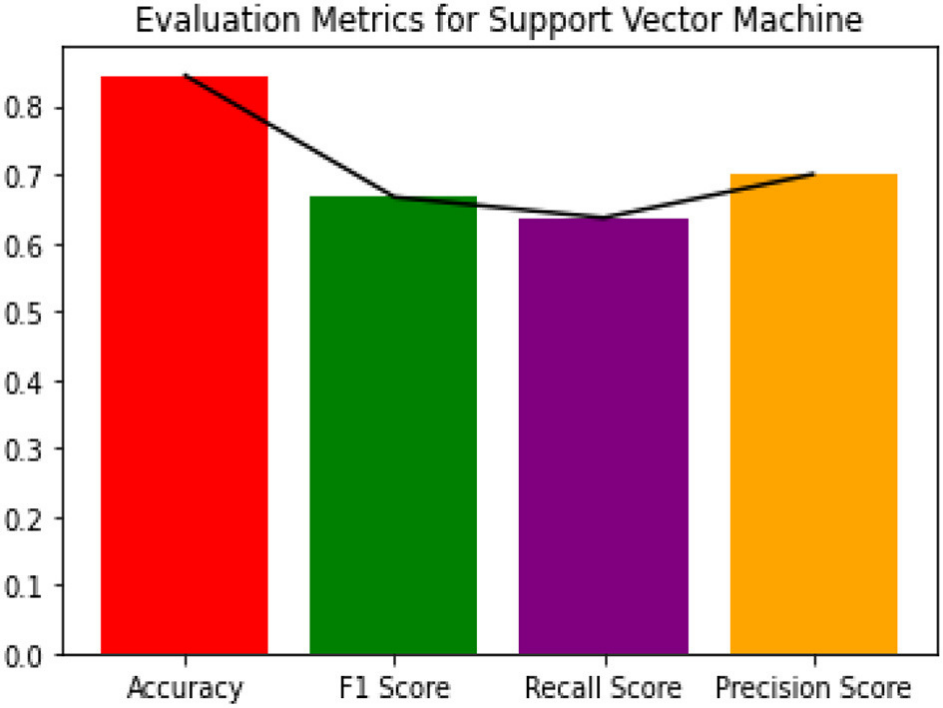
Evaluation metrics for SVM classifier.

**Figure 5:**
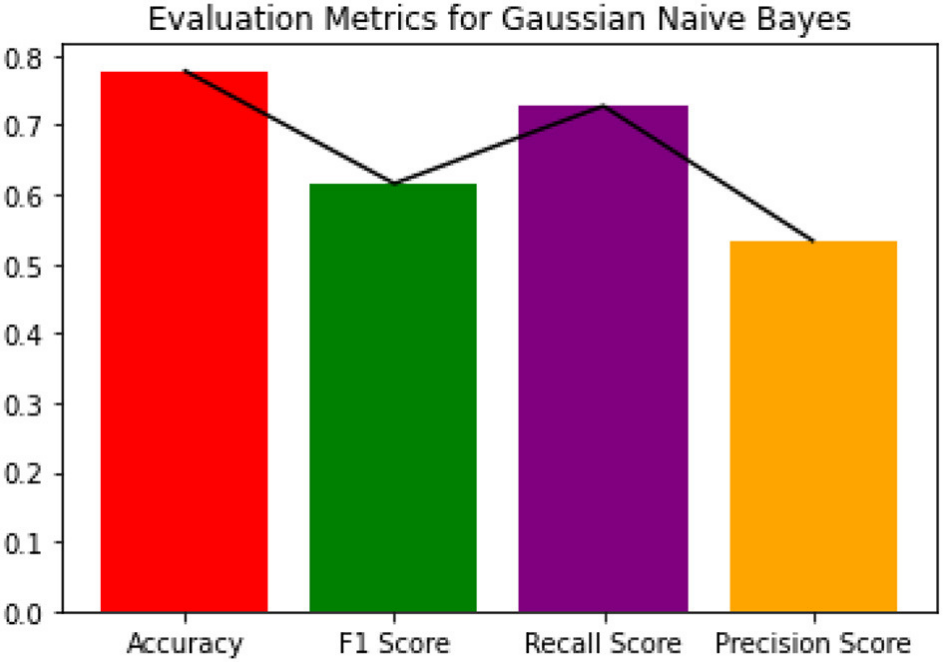
Evaluation metrics for Gaussian NB.

**Figure 6:**
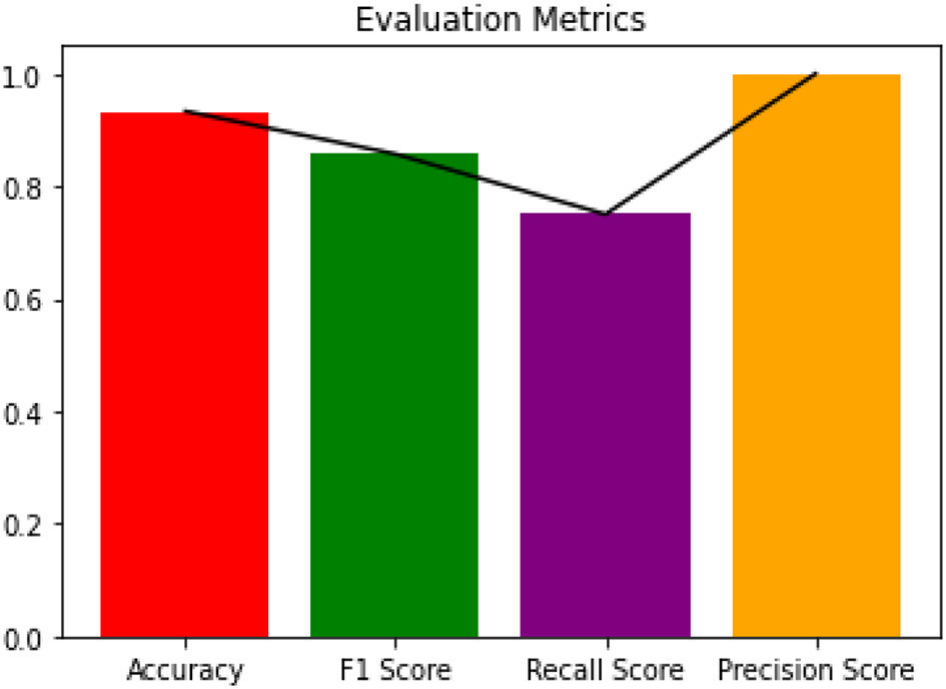
Evaluation metrics for Boosted Random Forest.

Since the dataset we used can be an imbalanced dataset, we will be using F1 Score as the primary metric for comparison. Figures 3–6 shows the model performances for all the models stated above.

**Figure 7:**
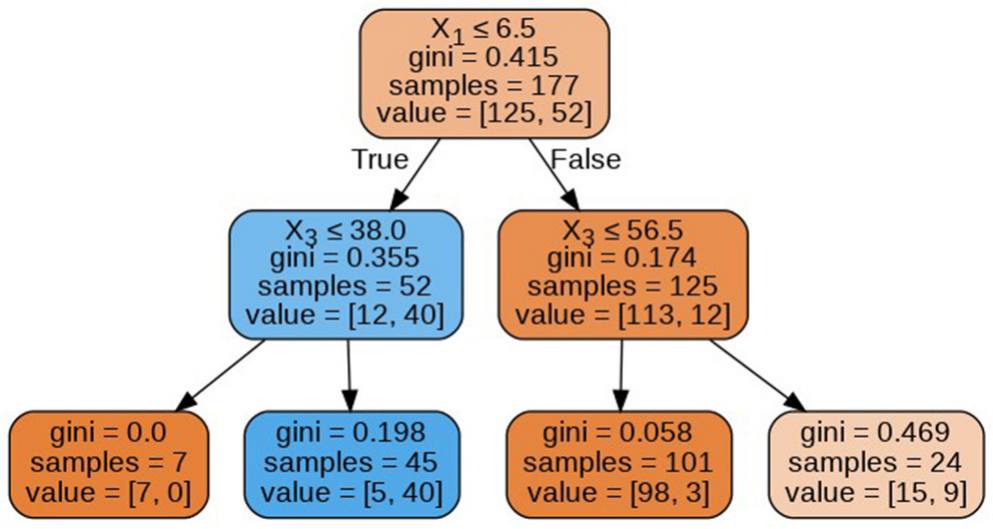
Decision tree.

The decision tree constructed for estimating the target variable is visualized in Figure 7. The decision tree has a depth of 2 and the Gini index of every node is <0.5, which indicates an imbalance in the training data.

Since Boosted Random Forest algorithm is the best performing model, we will fine tune the model for better performance on the dataset.

## Discussion

### Boosted Random Forest Classification

A Boosted Random Forest is an algorithm, which consists of two parts; the boosting algorithm: AdaBoost and the Random Forest classifier algorithm (27)—which in turn consists of multiple decision trees. A decision tree builds models that are similar to an actual tree. The algorithm divides our data into smaller subsets, simultaneously adding branches to the tree. The outcome is a tree consisting of leaf nodes and decision nodes. A decision node has two or more branches representing the value of each feature (like age, symptom1, etc.) tested and the leaf node holds the result value on the patient's prospective condition (target value).

Multiple classifier decision trees (ensemble of classifiers) eliminate the risk of failure of a single decision tree to correctly predict the target value. Thus, the random forest averages the result provided by multiple trees to provide the final result.

The margin function for the random forest is expressed in Equation (5), the generalization error in Equation (6), and confidence in the prediction in Equation (7). Here *h*_1_(*x*), *h*_2_(*x*), …, *h*_*k*_(*x*) is the ensemble of classifiers (decision trees) and the training data is drawn from the vectors *X, Y*.

The margin function is expressed as follows:

(5) $$\begin{array}{r} \text{mg }\left(\text{X},\text{ Y}\right)=\text{a}{\text{v}}_{\text{k}}\text{I}\left({\text{h}}_{\text{k}}\left(\text{X}\right)=\text{Y}\right)-\text{ma}{\text{x}}_{\text{j}\neq \text{Y}}\text{a}{\text{v}}_{\text{k}}\text{I}\left({\text{h}}_{\text{k}}\left(\text{X}\right)=\text{j}\right) \end{array}$$

where the indicator function is denoted by *I*(.). The generalization error is given as follows:

(6) $$\begin{array}{r} \text{P}{\text{E}}^{*}={\text{P}}_{\text{X},\text{Y}}\;(\text{mg }\left(\text{X},\text{ Y}\right)<0) \end{array}$$

where the probability is expressed over the *X, Y* space. In random forests, we have *h*_*k*_(*X*) = *h*(*X*, Θ_*k*_), therefore the number of classifiers (decision trees) increases, for all the sequences of trees. The probability PE^*^ converges to Equation (7), from the Strong Law of Large Numbers and tree structure.

(7) $$\begin{array}{r} {\text{P}}_{\text{X},\text{Y}}\left({\text{P}}_{\text{Θ}}\left(\text{h}\left(\text{X},\text{Θ}\right)=\text{Y}\right)-\text{ma}{\text{x}}_{\text{j}\neq \text{Y}}{\text{P}}_{\text{Θ}}\left(\text{h}\left(\text{X},\text{Θ}\right)=\text{j}\right)<0\right) \end{array}$$

Applying the boosting algorithm AdaBoost (28) provides a corrective mechanism to improve the model after every prediction of patient state. Eventually, the decision is a result of summing up of all the base models. It is one of the most efficient techniques in ML.

The corrective mechanism can be expressed as follows Equation (8). Given (*x*_1_, *y*_1_), …, (*x*_*m*_, *y*_*m*_), *where x*_*i*_ ∈ *X, y*_*i*_ ∈ *Y* = {−1, +1}. For, *t* = 1, …, *T*. Initialize $D_{1}\left(i\right)=\;\frac{1}{m}$. After training a weak learner, random forest in our case, using distribution *D*_*t*_.

Get the hypothesis, *h*_*t*_ : *X* → {−1, +1},

With the error *e*_*t*_ = *P*_*r*_*i*~*D*_*t*___ [*h*_*t*_ (*x*_*i*_)≠ *y*_*i*_]

After choosing $\alpha_{t}=\;\frac{1}{2}ln\;ln\;\left(\frac{1-e_{t}}{e_{t}}\;\right)$

Update: $D_{t+1}\left(i\right)=\;\frac{D_{t}(i)}{Z_{t}}\times \{e^{-\alpha t}\;if\;h_{t}(x_{i})=y_{i}\;e^{\alpha t}\;if\;h_{t}\left(x_{i}\right)\neq y_{i}=\;\frac{D_{t}\left(i\right)\;exp(-\alpha_{t}y_{i}h_{t}(x_{i}))}{Z_{t}}$

Here, *Z*_*t*_ is a normalization factor. We get the final hypothesis as follows:

(8) $$\begin{array}{r} H\left(x\right)=sign\left(\overset{T}{\underset{t\;=\;1}{\sum }}\alpha_{t}h_{t}\left(x\right)\right) \end{array}$$

Here the dependent variable was the patient state (dead/recovered) while the independent variables were location, country, vis_wuhan, from_wuhan (hosp_vis—sym_on), age, gender, symptom (1–6). We have used the boosted random forest because of its accurate classification performance on imbalanced datasets (25, 29).

**Figure 8:**
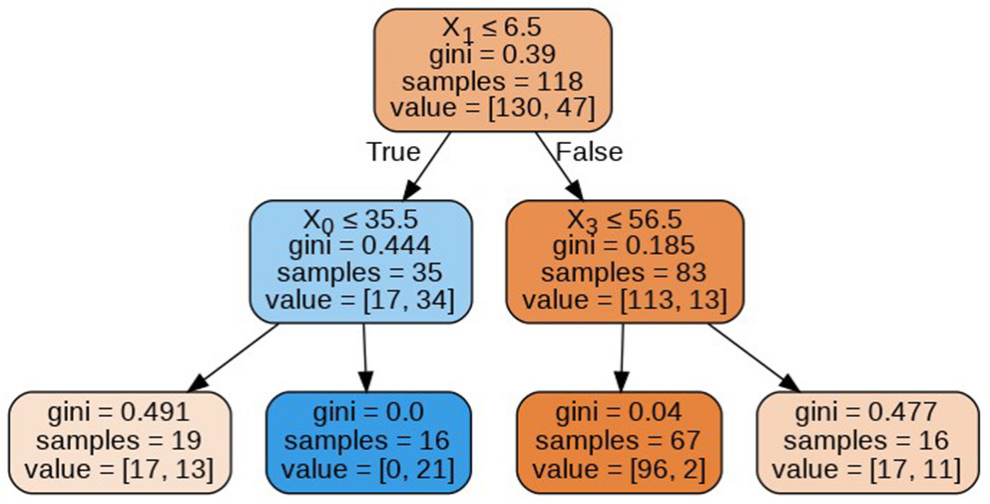
Decision tree 1.

**Figure 9:**
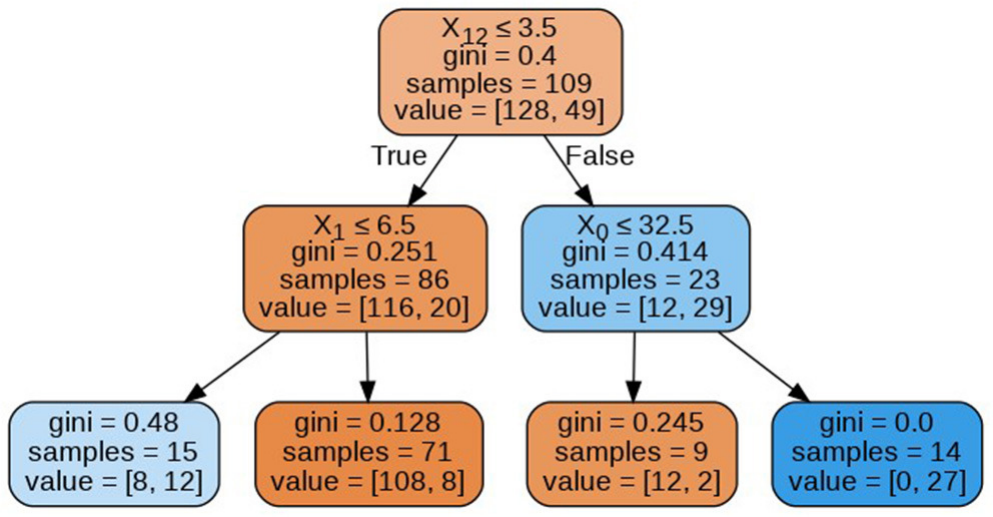
Decision tree 10.

**Figure 10:**
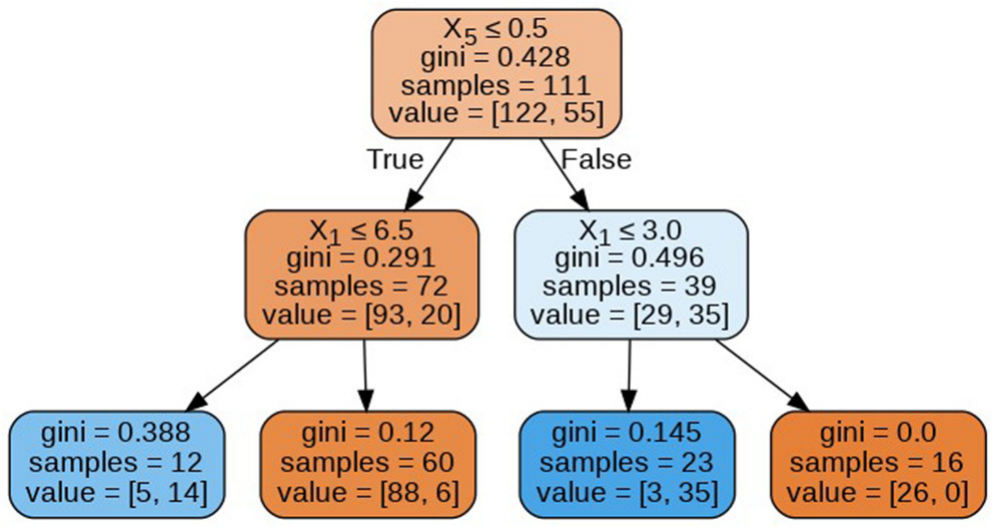
Decision tree 25.

**Figure 11:**
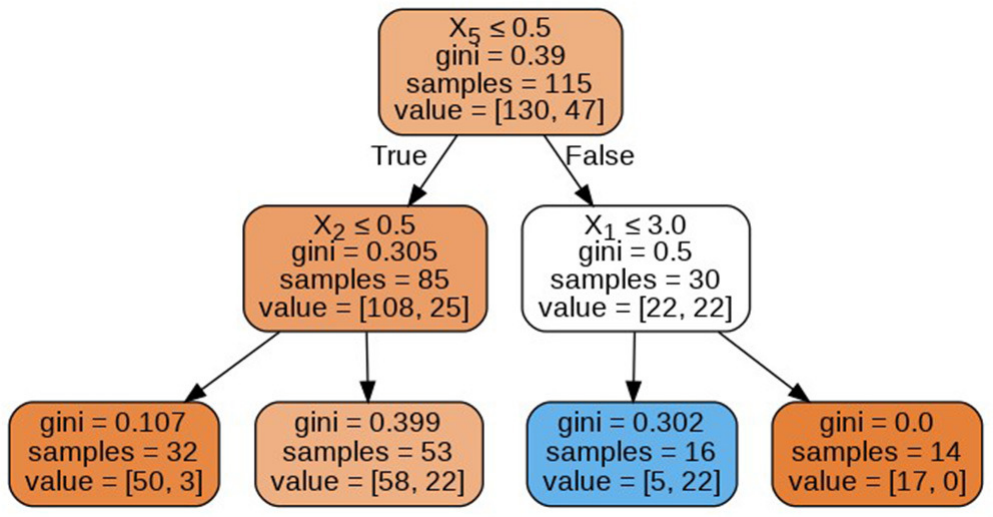
Decision tree 100.

The decision trees visualized in Figures 8–11 have a depth equal to two. Also, the Gini index in all the leaf nodes of all the trees is <0.5, which indicates the training dataset is imbalanced. Hence, for optimizing the performance of the model we have reduced the depth of trees to 2 and increased the number of estimators (decision trees) in the random forest to 100. This prevents high variance in the model and provides accurate predictions.

### Hyperparameter Optimization

Since the Boosted Random Forest Classifier was implemented using the default parameters, for the optimal performance of the model, we conducted a grid search over a grid of chosen parameters to gain a set of best performing parameters. We implemented the grid search using the GridSearchCV() function from Sklearn library. Table 2 presents the hyperparameters as returned by the grid search algorithm (30).

**Table 2:** Optimal hyperparameters returned by grid search.

**Parameters**	**Value**
n_estimators	100
max_depth	2
min_samples_leaf	2
min_samples_split	2
criterion	gini

Table 3 presents the evaluation metrics of the Fine Tuned Boosted Random Forest.

**Table 3:** Evaluation results.

**Metric**	**Score**
Recall score	0.75
Precision score	1.0
F1 score	0.86
Accuracy	0.94

**Figure 12:**
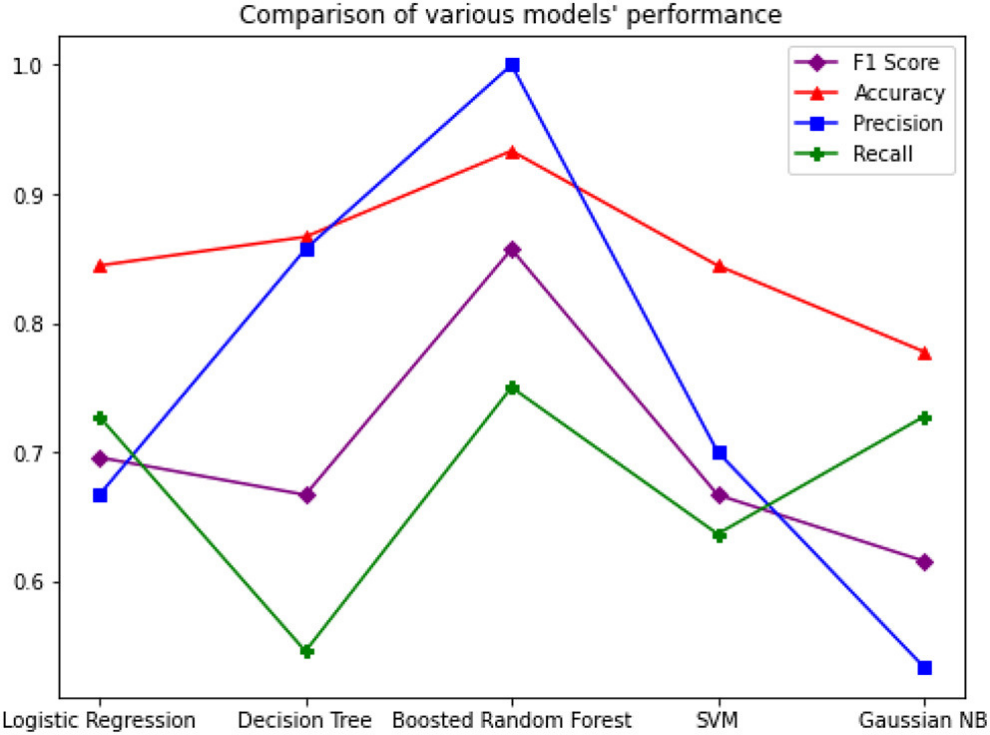
Comparison of Models' performance.

The study shows that Boosted Random Forest performs better while predicting COVID-19 patient deaths. Figure 12 graph compares the performance of all the models including Boosted Random Forest.

## Conclusion and Future Work

The application of Artificial Intelligence is very crucial to process patient data for efficient treatment strategies. In this paper we presented a model that implements the Random Forest algorithm boosted by the AdaBoost algorithm, with a F1 Score of 0.86 on the COVID-19 patient dataset. We have discovered that the Boosted Random Forest algorithm provides accurate predictions even on imbalanced datasets. The data analyzed in this study has revealed that death rates were higher amongst the Wuhan natives compared to non-natives. Also, male patients had a greater death rate compared to female patients. The majority of affected patients are aged between of 20 and 70 years.

Future work will focus on creating a pipeline that combines CXR scanning computer vision models with these types of demographic and healthcare data processing models. These models will then be integrated into applications that will support the growth of mobile healthcare. This can provide a step toward a semi-autonomous diagnostic system that can provide rapid screening and detection for COVID-19 affected regions and prepare us for future outbreaks.

## Data Availability Statement

The datasets presented in this study can be found in online repositories. For the reproducible code, please check out the GitHub repository: https://github.com/Atharva-Peshkar/Covid-19-Patient-Health-Analytics.

## Author Contributions

AP, CI, and RM: conceptualization. AP and RM: methodology, investigation, data curation, and writing—original draft preparation. AP, RM, SP, OJ, and NP: software. RS and JC: validation and visualization. CI, RS, and JC: formal analysis. AP, AB, and RM: resources. JC and CI: writing—review and editing, supervision. AB, AP, RM, SP, NP, RS, CI, OJ, and JC: project administration. All authors have read and agreed to the published version of the manuscript.

## Conflict of Interest

The authors declare that the research was conducted in the absence of any commercial or financial relationships that could be construed as a potential conflict of interest.
